# “Everything is Different:” Exploring Changes to Neighborhood Context, Physical Activity, and Food Behaviors Among Immigrants Settling in Emerging U.S. Latino Communities

**DOI:** 10.1007/s40615-026-02893-4

**Published:** 2026-03-07

**Authors:** Áine O’Connor, Katrina S. Edmond Taylor, Liliana De La Garza, D. Carolina Andrade, Deborah Salvo, Carrie R. Howell

**Affiliations:** 1 Department of Health Behavior, Gillings School of Global Public Health, University of North Carolina, Chapel Hill, NC, USA; 2 People, Health, and Place Unit, Prevention Research Center, Brown School, Washington University, Saint Louis, MO, USA; 3 Heersink School of Medicine, Department of Medicine, Division of General Internal Medicine and Population Science, University of Alabama, Birmingham, AL, USA; 4 Division of Public Health Sciences, Department of Surgery, Washington University School of Medicine, St. Louis, MO, USA; 5 Center for Diabetes Translation Research (WU-CDTR), Washington University, St. Louis, MO, USA; 6 Center for Research to Community Impact, College of Education, The University of Texas, Austin, TX, USA; 7 People, Health and Place Lab, Department of Kinesiology and Health Education, College of Education, The University of Texas, Austin, TX, USA

**Keywords:** Latino, immigration, neighborhood context, chronic disease, qualitative

## Abstract

**Background:**

United States (U.S.) Latinos face high risks of chronic conditions such as diabetes and cardiovascular disease. Foreign-born Latinos in nontraditional immigrant-receiving communities (“emerging Latino communities”) may face unique barriers to engaging in healthy behaviors that can prevent disease. Upon arrival to the U.S., immigrant Latinos experience changes to their built and social environments that alter eating habits, social networks, physical activity options, and access to care in ways that may be unique in areas with small Latino populations. The Context Matters study in St. Louis, MO and Birmingham, AL aimed to characterize these changes to daily context and identify the mechanisms behind changes to health behavior after immigration.

**Methods:**

From September 2022 – April 2024, we conducted semi-structured interviews with service providers to local Latino communities in St. Louis and Birmingham and interviews as well as facilitated focus groups with Latinos who had immigrated to either city within ten years. Using structural vulnerability and ‘necessity versus choice-based physical activity models’ frameworks, we developed interview and focus group guides to ask participants about changes to context and daily living following immigration from Latin America. We used a hybrid deductive-inductive approach for thematic analysis.

**Results:**

Across both study sites, we conducted 21 interviews with community service providers and facilitated focus groups or interviews with 25 Latino immigrants. Participants described common differences between Latin American places of origin and the two emerging communities: in active living opportunities, feelings of neighborhood cohesion, and food and healthcare environments. Social class and norms additionally informed views on physical activity, transportation, diet, and care-seeking.

**Conclusions:**

This study provides additional depth to literature on chronic disease prevention among immigrant Latinos by identifying unique challenges to healthy behavior in emerging Latino communities. Our results support health lifestyles theories, finding that social class, economic stratification, place, and culture are necessary constructs for understanding changes to individual health behavior. Future research in emerging immigrant communities may benefit from assessing how patterns of health behaviors vary among subgroups and measuring environmental, social, and political contexts longitudinally and at multiple levels of effect.

## Background

Contextual factors such as built environment, social environment, and access to healthcare are known determinants of health behaviors like physical activity and healthy eating, which are in turn associated with incidence and outcome risks for noncommunicable and chronic diseases [1–3]. In the United States, people of Latin American origin or ancestry (hereafter, “U.S. Latinos”) face high risk for chronic conditions such as diabetes, cardiovascular disease, and overall poor metabolic health [4, 5]. Such risks may be compounded for immigrant Latinos—those born outside of the 50 U.S. states and Washington, D.C. in places such as Mexico, Puerto Rico, Bolivia, Colombia, etc.—who face additional barriers to accessing community services, healthcare, and healthy places to live [6, 7].

Latin American immigrants to the United States exhibit an initial health advantage over non-Hispanic whites, despite lower average socioeconomic status and higher rates of some risk factors for chronic disease (i.e., the Hispanic immigrant paradox) [8–10]. At the individual level, this advantage may degrade as an immigrant’s time living in the U.S. influences activity and diet habits initially developed in Latin American places of origin [9, 11]. At the community level, sharp changes to the built and social environment from a Latin American place of origin to a U.S. setting may fundamentally alter patterns of daily living, including food shopping and availability, transportation modes, social activity, and access and use of healthcare services, all of which have consequences for health and health behavior [3, 12].

Studies measuring the effects of migration-induced context change as a determinant of Latino immigrant health have largely focused on areas with historic Hispanic and Latino populations (e.g., U.S.-Mexico border states, Florida, New York, etc.) [11]. Even in those contexts, the effects of context change appear mixed and the causal pathways, complex. Living in neighborhoods with higher proportions of Latin American immigrants can be associated with a lower likelihood of consuming unhealthy foods among Latinos, but also reduced physical activity, perceptions of neighborhood quality, and social cohesion [13]. Other studies of Latino health in place report similarly contradictory relationships between diet quality, physical activity opportunity, and social environments when controlling for individual-level factors like socioeconomic status and acculturation [11]. Further, determinants of healthy lifestyles among immigrants to traditional U.S. Latino settings may not accurately reflect obstacles to healthy behavior in socially and geographically fragmented *emerging communities*, where U.S. Latino populations are experiencing new growth [14–16]. The effects of this dispersion are undermeasured and important to understanding health outcomes, as immigration to places with low Latino populations is associated with poor social capital, social support, and political visibility, hindering advocacy and resource acquisition to improve immigrants’ neighborhoods and access to care [14, 15, 17].

Understanding the variety of changes to neighborhood context that occur during migration, and the individual responses to these changes, is necessary to inform interventions for disease prevention and management [12, 14]. There is little research, however, on the ways that Latin American immigrants’ patterns of health behavior fluctuate with the significant changes to the social, political, and physical environment that occur with migration to emerging Latino communities. By eliciting narrative data, qualitative methods can overcome some of the logistical and methodological challenges of assessing behavior change over time and across borders [11, 18]. We present the results of interviews and focus groups conducted in emerging Latino communities in Birmingham, Alabama and St. Louis, Missouri as part of the mixed-methods Context Matters pilot study. Context Matters aimed to document changes in community-level factors experienced by new Latino immigrants in Missouri and Alabama, and to examine how these major context changes influenced health behaviors.

## Methods

### Parent study & setting

The Context Matters study utilized field-based surveys and qualitative methods to explore the feasibility of gathering data on the impact of context change (from country of origin to setting of arrival in St. Louis or Birmingham) on health behaviors, particularly physical activity, diet, and care-seeking, among recent Latin American immigrants in emerging Latino communities. We conducted interviews and focus groups with participants from three counties each within the St. Louis and Birmingham metropolitan statistical areas that have experienced recent growth in foreign-born Latino populations (Table 1).

### Participant eligibility & enrollment

#### Service providers and community advocates

Adult (19 years of age or older) members of Latino-serving community organizations, advocacy groups, and/or businesses comprised our interview sample. We recruited potential participants by conducting organizational landscape analyses, using Internet searches of local news, maps, and chambers of commerce to identify eligible enrollees. Researchers contacted participants via publicly available contact information (email and telephone) to share study and consent information, enroll, and schedule one-on-one interviews. We invited enrolled service providers to share study information with colleagues meeting inclusion criteria.

#### Latin American immigrants

Adult (19 years or older) residents of selected metropolitan counties in St. Louis and Birmingham who had lived in the United States for less than 10 years and in the metropolitan area for at least 6 months were also eligible to participate. We recruited these participants using flyers online and in high-traffic physical spaces; in-person recruiting in public spaces and community events; and collaboration with community organizations who consented to share study flyers via e-newsletter. Participants reviewed consent documents and enrolled by providing contact information to a secure REDCap form or contacting study staff using the contact information on our flyer.

#### Qualitative approach

We used a hybrid inductive/deductive approach to qualitative data analysis, aiming for thematic saturation [19]. The hybrid approach was best suited to the study’s nesting of exploratory qualitative research aims inside an experimental feasibility test (the overarching Context Matters pilot). With regards to the deductive component, data collection guides and initial codebooks were structured based on previous work by our team and others [20–23], and were grounded on two key conceptual frameworks that question the notion of individual-level choice making, or agency, among socially vulnerable groups: structural vulnerability and the ‘necessity versus choice-based physical activity models’ frameworks [24, 25]. Meanwhile, inductive analysis of participant narratives informed the identification of most analytic themes during coding.

Teams at each site included bilingual, Latino researchers and longtime residents of St. Louis and Birmingham. Researchers consulted on a biweekly basis to troubleshoot data collection and analysis and determine thematic saturation. Some data collectors shared or could relate to participants’ experiences with immigration to emerging Latino communities. All data collectors received training in inductive, qualitative techniques and use of the interview and focus group guides.

### Data collection

We created separate interview and focus group guides for the two participant populations, with questions derived from investigators’ previous work in Latino health and contextual determinants of chronic disease [20–22]. Service providers and community advocates were invited to interviews to share their perceptions of how changes to environmental and social contexts upon immigration to St. Louis or Birmingham might impact social engagement, physical activity, diet, and overall quality of life among recent Latino arrivals.

Latino immigrant participants joined focus groups, where they described daily life in their place of origin and current neighborhood. Data collectors probed groups to share experiences with changes in neighborhood composition or layout, physical activity, social connections, diet, and quality of life. Due to scheduling challenges at the Birmingham site, that research team pivoted to conducting individual, semi-structured interviews with Latino immigrant participants using the study focus group guide.

We collected data from September 2022-April 2024 in St. Louis and November 2022-April 2024 in Birmingham. Data collection was conducted in English (*n* = 20 service providers, *n* = 0 Latino immigrants) or Spanish (*n* = 1 service provider, *n* = 25 Latino immigrants), depending on participant preferences. To alleviate scheduling and transportation barriers to participation, we recorded interviews and focus groups using Zoom teleconferencing. Participants received $50 gift cards upon completion of interview or focus group sessions.

### Analysis

We used professional, human services to transcribe and translate de-identified audio recordings. All researchers received training in best practices for inductive analysis [26] and thematic synthesis [27]. Two bilingual researchers developed a preliminary codebook a priori based on interview and focus group guides (e.g., creating general codes for “physical activity,” “diet,” “neighborhood change,” etc.) and added additional codes and themes after independent, inductive analysis of one interview and one focus group transcript. Two other researchers refined the resulting pilot codebook by individually and consensus-coding one interview and one focus group transcript from each study site. Researchers from both study sites finalized the codebook prior to analysis, including edits by Latino researchers to ensure codes captured culturally and linguistically specific themes after translation.

Using the revised codebook, a six-member team individually and consensus-coded all focus group and interview transcripts using NVivo v.14 [28]. Coding pair assignments were made using a random number generator; every transcript received two coders. Throughout analysis, coders met and corresponded to iteratively confirm themes and interpretations. Following consensus coding, a four-person subset of the coding team produced thematic summaries for each code family, including exemplary quotes, which all coders reviewed.

## Results

### Participant characteristics

We conducted nine interviews with service providers and community advocates in Birmingham and twelve in St. Louis for a total sample of 21. All but one of the Birmingham service providers (*n* = 8, 88.9%) were born and/or had grown up in Latin American places of origin compared to 58.3% (*n* = 7) in St. Louis. Most were from Mexico (*n* = 4, 19.0%), Puerto Rico (*n* = 4, 19.0%), or Colombia (*n* = 3, 14.3%). All service providers reported working or volunteering at service-based, institutional, or community organizations focused on the local Latino community.

Twenty-five Latino immigrants participated across both study sites. We enrolled six St. Louis participants each in two focus groups for a total of 12. In Birmingham, 13 Latino immigrants completed semi-structured interviews with research staff using the focus group facilitation guide. Altogether, this group represented twelve different places of origin, most frequently Honduras (*n* = 6, 24.0%), Mexico (*n* = 4, 16.0%), El Salvador (*n* = 3, 12.0%), and Colombia (*n* = 3, 12.0%). Figure 1 shows the distribution of places of origin across study sites and participant populations.

### Thematic analysis

#### “We are not a monolith:” Differing health-related needs and experiences within emerging Latino communities

Participants represented a variety of lifestyles, behaviors, and experiences in places of origin and in their new United States communities. Service providers and advocates indicated that this diversity of cultural and national backgrounds influenced diabetes-related health behaviors in meaningful ways. As a St. Louis service provider noted early in their interview,

I just think that it’s important when you talk about doing a study in the Latino community that you understand that we understand that we are not a monolith. That there are a bunch of different countries represented and languages and even cultures within us. I know we get lumped together a lot of times.

– St. Louis service provider.

Perceived changes to neighborhood environment were heavily dependent on where a participant had lived prior to settling in Birmingham or St. Louis. For those coming from major metropolitan areas like Mexico City, these cities often seemed smaller, containing fewer resources for healthy living than their place of origin. Others migrating from rural areas of Latin America mentioned major differences in urban layout (i.e., sprawl), transportation (e.g., road networks and vehicle transit), and amenities for daily life.

The small size and frequent reconstitution of emerging Latino communities produced a sense of instability that hindered the creation of supportive social networks. Competent and culturally representative community organizations were important to maintaining and supporting community growth. Some immigrant participants indicated that exposure to other, highly educated Latinos working in support organizations could make them feel less alone. Service providers, however, shared challenges to addressing community needs among heterogeneous Latino populations. Immigrants had to develop support networks with others who did not share their national or cultural backgrounds.

…there’s always this sort of blank assumption that because we share the language, we can share everything else—including some very personal needs, right? But in reality, what happens is you feel almost as alone [in the Latino community] as you did in the wider community—because yes, they’re closer to your people, but they’re not really your people.

– St. Louis service provider.

#### Social networks and service provider resources play large roles in determining adjustment to emerging communities

Most immigrant participants reported having someone to turn to in times of need but differed in how and where they found social support. Cultural divergences between Latino immigrants and American neighbors were a source of some bemusement. One Birmingham immigrant characterized Americans as nice but reserved, saying, “they lock themselves in their homes” compared to highly involved neighbors in Nicaragua. Some agreed that American friendliness felt largely symbolic—“smil[ing], acting welcoming”—and did not associate this with social support. Multiple participants therefore reported feeling more alone in the United States than in their countries of origin.

Immigrants were able to find local resources by using social capital, asking family, friends, or acquaintances where and how to find healthcare, social benefits, and food assistance. Those with family in the U.S. reported that familial support accelerated adaptation to life in Birmingham or St. Louis, while those who migrated alone had to be more intentional about developing networks. Many reported building social capital with other Latinos in public places and events such as festivals, restaurants, churches, clubs, sports, and community organizations. While these community spaces were perceived to be widely available, multiple participants mentioned not having enough time or money to use them as they wished. Others noted that building community in their emerging Latino community was more difficult, and potentially painful, compared to their place of origin.

…although I have found community with other people, the way it is done in Puerto Rico is – I don’t know, different….[In Puerto Rico] it was kind of easy to find community and live music and a place where you can like, you don’t have to know someone to have a good time. Here it’s like, how difficult it is to have that place or to find that place and also, well, if sometimes you found it, you also see racism….

– St. Louis immigrant from Puerto Rico.

*Chisme* (gossip) was a frequently cited factor in adjustment to the U.S. and in decision-making regarding healthcare and health behavior. While *chisme* is a common social practice in many Latino communities and has potential benefits in solidarity-building and knowledge-sharing [29, 30], participants identified several ways chisme presented barriers to healthy behavior and wellbeing in their community. In particular, the possibility that violations of social or cultural norms could be exposed to social networks through *chisme* was a meaningful barrier to care. One service provider noted that *chisme* made it difficult for their Latino clients to leave dangerous situations like abuse, or to contemplate big changes to family life, out of “worry about what others in the community might think of their actions.” Fear of *chisme* kept one participant from going to “clinics” for care out of fear that people would know they were “broke.” There was a prevailing sense that, because of the small size of the Birmingham and St. Louis Latino communities, all Latino people knew each other, and this perceived lack of privacy exacerbated fears of social consequences for violating norms.

Keeping social ties intact was reported to be particularly important due to the presence of language barriers in emerging communities, making institutional navigation of resources difficult for many. Community organizations were necessary assets for gaining familiarity with federal, state, and especially local institutions, as many metropolitan resources lacked Spanish language infrastructure that might be observed in more historically Latino communities. A service provider working in small business support narrated receipt of frequent calls from help-seeking immigrants hoping to navigate the structures of daily life—“how to request a loan, where to go to buy a house, what kind of [health] insurance they need”—indicative of a consistent need for holistic resource navigation, which many community organizations provided for immigrants even outside of their stated scope of work.

#### Car dependence and limited public transit in emerging communities are barriers to wellbeing

Participants frequently reported walking less in the U.S. compared to many places of origin, where they could walk or cycle to attend to basic needs like purchasing food or commuting to work. Asked about changes they had noticed in their neighborhood environment since immigrating, one Birmingham participant from Mexico laughed and said, “Everything is different. The street; the buildings; the parks; the stores, everything is different.”

Immigrants in both settings frequently mentioned public transportation as a facilitator of active transit, with some differences by place of origin. One participant indicated that where they lived in Mexico, “there is not much transportation; walking is used a lot.” In other places of origin, public transportation was reported to be more robust than either Birmingham or St. Louis and typically involved walking or cycling to buses and trains.

Those who discussed active transportation sometimes implicitly connected it to other health behaviors like healthy eating, noting that there was a “balance” possible in Latin American places of origin that seemed harder to achieve in the U.S.

We’re very active during the day [in Latin America], so we are not sedentary. But here, we adopt the American diet, which is also very high in carbs, but we have cars, or we stay at the office all day, and so we don’t burn the calories that we were burning before in South America.

– Birmingham service provider.

Access to a personal vehicle was perceived as essential to receiving health services, finding employment, and navigating the urban environment in emerging Latino communities. A Birmingham service provider described a scenario in which having even only one vehicle may not be enough for a family with “limited resources,” as each adult may need to separately go to work, purchase food, and care for children. For undocumented persons, however, difficulty acquiring driving licenses or auto insurance made driving a significant but necessary risk. Carpooling and ridesharing were common workarounds, with several reporting reliance on family, friends, or coworkers to get from place to place. Vehicle ownership was also identified as a new cultural expectation upon immigration to the U.S.: another service provider suggested that being “the only one walking” can make Latin American immigrants feel out of place—even stigmatized—in emerging Latino communities, as car ownership is “this sort of underlying, in many ways unwritten… piece of the American dream.”

#### Culture, social norms, and time determine physical activity and eating behaviors

Across participant groups, many noted cultural and class-based differences in norms around physical activity in Latin America, with St. Louis service provider describing leisure-time exercise as “an elitist opportunity.” These dynamics were corroborated by a Birmingham service provider saying that “physical activity in low-income families is not a thing,” and that people may be mocked for exercising. Instead, activity in places of origin was characterized by social and cultural networks that were hard to find in the small Latino communities of Birmingham and St. Louis. For instance, one service provider noted that preference for sports and activity “depends on your cultural background and how you were raised,” noting that popular activities vary between Latin American countries. A Puerto Rican focus group member shared, “It’s not like I can go to the park in my community in [St. Louis], and I’m going to find a group of people playing soccer. That’s not happening”—suggesting that even for those interested in recreational activity and with the time to seek it out, finding familiar and enjoyable activities was challenging.

While leisure (free time) physical activity was often seen as more accessible and expected in U.S. contexts, with participants commonly identifying access to facilities like gyms and parks, leisure time to engage in activity was reported to be in short supply. Many immigrants indicated that spending more time working and commuting in the U.S. than in places of origin limited time for leisure physical activity. Immigration into the American work culture seemed to affect participants’ perceptions of leisure time regardless of the type of work they conducted: while occupational labor was often a primary source of physical activity, participants with other types of jobs also referenced exhaustion, heavy workloads, and a dearth of time for recreation and exercise. Likewise, commuting was a common source of physical activity in their home countries, while in the U.S. most of it takes place by car or motor vehicles. A Birmingham immigrant originally from Cuba described how pressure to be “always working” limited engagement in healthy behaviors in leisure time, such that “when you are free, what you want to do is rest.” Service providers in both St. Louis and Birmingham corroborated these findings, sharing that many Latino immigrants accepted labor-intensive jobs, e.g., cleaning, food service, construction, painting, and gardening or lawncare.

Time was also a limiting factor for healthy eating. Due to the time and energy costs of work, some reported resorting to fast food and readymade meal options. Cultural practices of connecting with family and friends over meals that were important in places of origin felt no longer feasible in the context of American work and school schedules.

In Cuba you work, but you don’t live off work….But you have more time and you can do physical activities. In Cuba you can dance, you can exercise, but the bad thing is what I explained to you. Here you can do physical activity, but you don’t have the time.

– Birmingham immigrant from Cuba.

#### Low availability of familiar foods influences dietary habits

Along with challenges finding time to eat socially, participants reported difficulty finding culturally relevant foods. Expressions of grief and wistfulness for beloved dishes were common. There weas widespread agreement that Latino food in immigrants’ communities was frequently limited to Mexican ingredients, dishes, and restaurants more familiar to American consumers, reflecting a perception of Latin American cuisine defined by “tacos and tamales.” Grocery stores in areas with scattered, diverse, and relatively small Latin American populations were unlikely to stock culturally relevant foods for all; commonly referenced missing ingredients included spices, fresh fruits for making juices, recao (culantro, used in Puerto Rican sofrito), particular types of ajís (peppers), and plátanos (plantains).

Immigrants frequently reported that the quality of food available to them in the U.S. was poorer than in Latin America. American food was often perceived as fundamentally unhealthier than Latin American food, e.g., fattier, heavier, made with preservatives, fast or premade for convenience, and farmed with chemical pesticides. Service providers indicated that the “shock” of unfamiliar ingredients and products had health consequences for recent Latino/e arrivals:

…you go to the supermarket and you pick up a food label here to try to understand it [in] a language that’s not your preferred language, assuming you can read it–and you’re like, ‘I can’t do this…I can’t be spending 20 minutes trying to figure out what’s what.’ And so people kind of get into sort of, you know, what’s on sale. Or looks good on the packaging. And those are then habits that are hard to get out of, right? And you develop a palate for these things…for the way that food is made in the United States with high content of salt and sugar, and other not good things. And so it’s difficult then to break away from that.

– St. Louis service provider.

#### Use of healthcare services hinges on linguistic access and cultural competence

Language was a commonly noted barrier to managing health and wellbeing. Many immigrants expressed what a Birmingham service provider described as “a constant worry and concern” of not understanding their surroundings. Participants reported seeing little-to-no Spanish signage in public places and healthcare settings, limiting their knowledge of resources available for healthy living. Multiple immigrants reported avoiding healthcare out of fear of stigmatization from providers for speaking imperfect English. Service providers more readily identified the uncertainty of documentation status as a barrier to accessing care:

They prefer to be under radar. They don’t want to bring attention to themselves. So they just come to work, make money, send money to their relatives in their other countries. And that’s really what they’re here for. And a lot obviously, because if they’re here, depend[s] on their legal status….

– Birmingham service provider.

Poor access to care was a particular barrier to mental health. Beyond language, participants referenced cultural stigma around mental health—the “big taboo,” as one St. Louis service provider described it—as a determinant of care-seeking among immigrants. Because stigma around mental health could threaten immigrants’ social position—again, as a result of chisme among small social networks—access to professional support was reported to be important. Multiple participants described depression, loneliness, or anxiety upon arrival to Birmingham or St. Louis, but feeling ill-supported by service providers with whom they could not communicate. A St. Louis community advocate said that trying to find the vocabulary to discuss the “stress and in some causes trauma” of immigration in English increased anxiety around seeking help.

…let’s say [the patient] speaks English perfectly… they might mention something that has to do with how they were raised, or how their people act back home. And if the psychologist and the mental health specialist has no idea what that is, doesn’t know how to comment properly or makes some inappropriate [comment] without really realizing it…you know, know that you’re never going to see that patient again.

– St. Louis service provider.

## Discussion

The complete change to neighborhood, social, and cultural context that occurs during migration can wholly disrupt healthy habits and behaviors that protect against chronic disease and adverse mental health conditions. Our findings detail a multitude of changes Latin American immigrants experience in neighborhoods, social networks, employment options, and access to care upon relocation to emerging Latino communities in the United States. We found common differences between origin and settlement locations as settings for healthy behavior: in opportunities for active transit and physical activity, norms related to work, feelings of neighborhood cohesion, and access to resources for daily life—especially, for navigating local food and healthcare systems.

Further, we found that social class, economic stratification, and place and culture of origin were important determinants of these contextual factors among our participants. Perceived socioeconomic differences between Latin American places of origin and U.S. emerging Latino communities were often linked (implicitly and explicitly) to preferences and proscriptions on habits related to physical activity, healthcare-seeking, and transport. These findings support the theoretical conceptualization of health behavior patterns as *health lifestyles*, in which behaviors or habits reflect individual preferences that are shaped by that individual’s position within their social structures (and subject to social and structural determinants) [31–33]. In this case, the limited size of culturally homophilous social networks in our participants’ emerging communities seemed to make other key contextual differences—in expectations about work and leisure time, in opportunities for activity, in access to preferred foods and social engagements—feel deeper and more fixed.

For example, immigrants connected the challenges they faced in developing social support networks, accessing quality healthcare free of stigma, and finding preferred foods to the fact that the Latino community in their area felt small, and the community of people from their place of origin, even smaller. Participants identified barriers to healthy living in the lack of Spanish-language resources for area wayfinding, resource navigation, and other necessary information for daily life than we might find in more established Latino immigrant-receiving settings. Healthcare providers were perceived as less likely to speak Spanish, have translated materials, or understand culturally specific experiences, causing stress and worry among immigrants that in some cases led to abandoning professional health advice altogether. As suggested by structural vulnerability frameworks identifying undocumented Latino immigrants as uniquely at risk of ill health in the U.S., the precarity of documentation status was an additional limitation, with the need to “be under the radar” thought to preclude undocumented Latinos from care-seeking [25]. These results provide potential mechanistic explanations for previous studies documenting poor physical health and increased psychological stress, depression, and substance use among immigrant Latinos in emerging communities [15, 34, 35].

Differences in leisure time physical activity and in active versus sedentary travel modes between Latin American and U.S. settings had meaningful consequences for physical activity and healthy eating that seemed to be similarly defined by cultural differences and socioeconomic class status as much as individual preference. For those used to walking or using ubiquitous public transit in their place of origin to access employment, education, healthcare, and other basic services and opportunities, physical activity in the U.S. was often constrained to the “free time” domain (versus occurring as part of transportation), and was challenged by car-dependent built environments.

For those employed in manual labor in either setting, occupational physical activity was their dominant form of physical activity. In this, our findings support the utility of the “necessity vs. choice” frameworks for different physical activity types (recreational activity vs. active labor vs. active travel) for low-income and foreign-born workers in high-income countries, where limits to employment and mobility based on documentation status, licensing requirements, rejection of home-country qualifications, or socioeconomic status interact with neighborhood context to restrict choices for activity [24]. Our research also identifies additional limits to food availability (in the few stores stocking preferred ingredients for home cooking) and time to cook (due to the time demands of employment and commutes) among immigrants living in emerging Latino communities, potentially above and beyond the impact of those barriers to healthy eating among U.S. Latinos generally [23, 36].

This study’s primary limitation is its limited generalizability beyond the Latino immigrant communities of the St. Louis and Birmingham areas. The themes presented here may not represent the experiences of immigrants from other places of origin or emerging Latino communities. Because participant recruitment took advantage of networks of local service providers, some participants may have been more likely than other immigrants to feel community cohesion and support. Finally, to maximize comfort and build trust we elected not to collect participant information beyond place of origin and age of majority. This left us unable to catalogue differences in participant age, gender, health status, socioeconomic status or employment, neighborhood, and other characteristics that may have influenced responses. Future qualitative investigations might focus on the effects of neighborhood context change on health behavior in particular subpopulations.

The strengths of our study lie in its deep, novel assessments of neighborhood and social environments in two emerging Latino communities, which are understudied settings for place-based research on immigrant health [16, 37]. We benefited from the diversity of our participants, who shared many different cultures, socioeconomic statuses, immigration experiences, employment situations, and family structures. This heterogeneity enabled a nuanced evaluation of how immigration from Latin America to emerging U.S. communities interrupts and reconstructs health lifestyles among a highly varied group of individuals. While immigrant Latinos were our primary key informants for this study, service providers and community advocates—most of whom also identified Latin American places of origin—drew upon their experiences working with community members over time to fill information gaps, clarify local politics and social norms, and offer the institutional context necessary to our thematic analysis. While our analysis often found commonalities between the narratives presented by these two participant groups, it is necessary to note that service providers were often longtime residents of their area with institutional authority and employment at respected nonprofit and advocacy organizations, and thus were situated in their emerging communities in ways that may not be representative of recent Latino immigrants. In particular, the reported comments on documentation status and fear of deportation came from service providers, who may have been more comfortable describing the effect of immigration status on health behavior than actually undocumented participants.

This study provides many directions for future research to prevent, manage, and treat chronic disease in emerging U.S. Latino communities. For instance, in line with health lifestyles theory, our participants often described changes to their *patterns* of health behavior (e.g., linking physical activity with diet and care-seeking with social engagement), indicating that measuring health behaviors discretely may overlook important pathways of disease risk and prevention [32]. That behavioral patterns were often contextualized by participants’ employment status, place of origin, and language supports the use of structural vulnerability frameworks to differentially assess health impacts by economic and political status [25]. It also suggests that studies quantifying the effects of social and built environments on immigrant disease risk would benefit from assessing multilevel interactions with individual-level determinants of lifestyle such as socioeconomic status, employment, and place or culture of origin [4, 38]. Pattern analysis techniques, which can construct and operationalize health lifestyles as exposure variables with sociodemographic correlates, may enable more precise risk assessments within diverse populations [39, 40].

The variety of our participants’ experiences concur with literature suggest that longitudinal approaches are particularly relevant to the study of migration-related context change, as health and behavior vary by immigrants’ ages and stages of life [14, 41, 42]. Context change may have different effects on health behavior among those who undergo multiple migrations and according to the stress of the migration experience [12, 42]. Longitudinal, cohort-based, and phased study designs can compare contexts and behavior across places, stages of life, and neighborhood environments, but require extensive, international recruitment and participant tracking. As part of a separate study aim, the Context Matters study piloted the use of recall-oriented survey questionnaires using validated scales to measure health behavior (forthcoming) at place of origin and within emerging U.S. Latino settings, a promising, low-cost way of assessing perceived context change over place and time.

## Figures and Tables

**Fig. 1:**
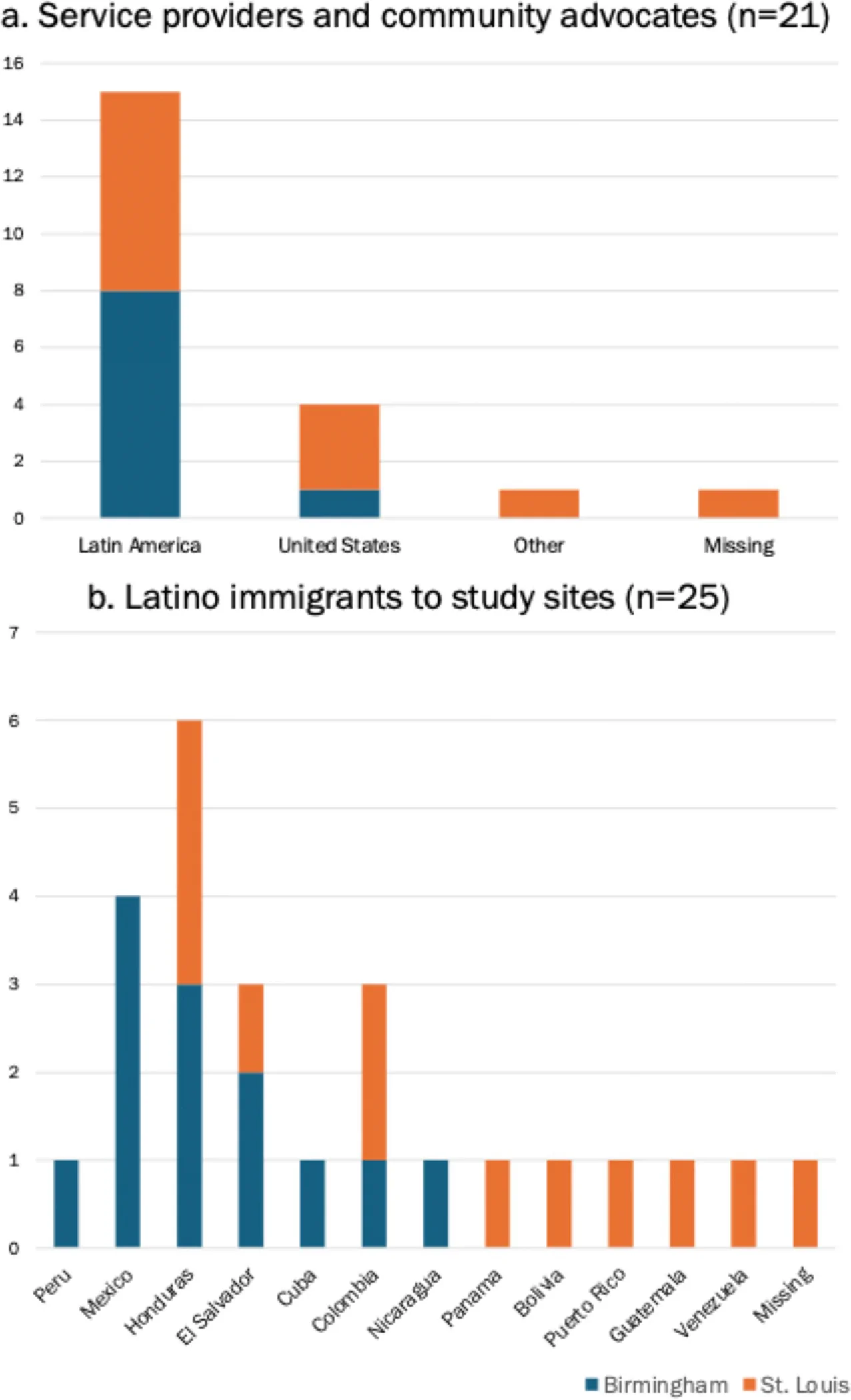
Participant place of origin by study site

**Table 1:** Top five places of origin of foreign-born Latinos by study metropolitan statistical area (MSA), 2021 (U.S. Census)

Birmingham Metropolitan Area		St. Louis Metropolitan Area	

Origin	Estimated foreign-born population (% MSA)	Origin	Estimated foreign-born population (% MSA)
Mexico	13,613 (1.15%)	Mexico	13,482 (0.62%)
Central America	3,646 (0.31%)	Central America	4,393 (0.20%)
South America	1,824 (0.15%)	South America	3,702 (0.17%)
Puerto Rico	1,381 (0.12%)	Puerto Rico	2,308 (0.11%)
El Salvador	1,363 (0.12%)	Colombia	1,523 (0.07%)

## Data Availability

The data that support the findings of this study are available on request from the corresponding author, D.S. Data are not publicly available to protect the privacy of study participants.
